# Segmentation of bones in magnetic resonance images of the wrist

**DOI:** 10.1007/s11548-014-1105-x

**Published:** 2014-08-06

**Authors:** Justyna Włodarczyk, Kamila Czaplicka, Zbisław Tabor, Wadim Wojciechowski, Andrzej Urbanik

**Affiliations:** 1 Jagiellonian University, Reymonta 4, 30-059 Krakow, Poland; 2 Cracow University of Technology, Warszawska 24, 31-155 Krakow, Poland; 3 Medical Center iMed24, Al. Jana Pawla II 41f, 31-864 Krakow, Poland; 4 Jagiellonian University Medical College, ul. Kopernika 19, 31-501 Krakow, Poland

**Keywords:** Image segmentation, Magnetic resonance imaging, Rheumatoid arthritis, Image registration, Atlas-based image segmentation, Watershed from markers, Wrist

## Abstract

**Purpose:**

Rheumatoid arthritis (RA) is a disease characterized by progressive and irreversible destruction of bones and joints. According to current recommendations, magnetic resonance imaging (MRI) is used to asses three main signs of RA based on manual evaluation of MR images: synovitis, bone edema and bone erosions. The key feature of a future computer-assisted diagnostic system for evaluation RA lesions is accurate segmentation of 15 wrist bones. In the present paper, we focus on developing a wrist bones segmentation framework.

**Method:**

The segmentation procedure consisted of three stages: segmentation of the distal parts of ulna and radius, segmentation of the proximal parts of metacarpal bones and segmentation of carpal bones. At every stage, markers of bones were determined first, using an atlas-based approach. Then, given markers of bones and a marker of background, a watershed from markers algorithm was applied to find the final segmentation.

**Results:**

The MR data for 37 cases were analyzed. The automated segmentation results were compared with gold-standard manual segmentations using a few well-established metrics: area under ROC curve AUC, mean similarity MS and mean absolute distance MAD. The mean (standard deviation) values of AUC, MS and MAD were 0.97 (0.04), 0.93 (0.09) and 1.23 (0.28), respectively.

**Conclusion:**

The results of the present study demonstrate that automated segmentation of wrist bones is feasible. The proposed algorithm can be the first stage for the detection of early lesions like bone edema or synovitis.

## Introduction

Rheumatoid arthritis (RA) is characterized by progressive and irreversible destruction of articular and periarticular structures. Studies of the epidemiology of RA show that about 0.5–1 % of a population suffers from RA, and an actual percentage of patients depends on gender, race/ethnicity and calendar year. It is estimated that in USA, over 1.5 million people suffer from RA and the costs of RA treatment are about 80 billion $ per year. The risk of death of people with RA is significantly higher compared with age- and sex-matched controls without RA mainly due to increased risk from gastrointestinal, respiratory, cardiovascular, infectious and hematologic diseases among RA patients [1].

In women, RA typically begins between ages 30 and 60, while in men, it often occurs later in life. The earliest symptoms of RA occur typically within the wrist joints [2]. The wrist joints are the most common anatomic location that is affected by RA. For this reason, an early diagnosis of RA within the wrist joints is essential for effective treatment of the disease. In 2005, the Outcome Measures in Rheumatology Clinical Trials MRI working group (OMERACT) developed an MRI-based scoring system (RAMRIS) [3] for assessing three types of RA-related lesions seen in MR images of the wrist: synovitis, bone edema and bone erosions. Importantly, synovitis and bone edema are lesions which precede irreversible joint destruction resulting in functional disabilities at later stages of RA, primarily cortical bone erosions and joints narrowing [4, 5] and thus assessing these lesions is of special importance for early diagnosis. Currently, these inflammatory changes can be detected only in MR and ultrasound images, but for the latter case, no diagnostic standard exists.

Since its recommendation, it has been shown that RAMRIS is reproducible and sensitive to changes due to therapy or disease progress. However, some of its limitations have been also recognized. Firstly, RAMRIS scoring is time-consuming since it requires analyzing and quantifying multiple, frequently small details in three dimensions (e.g., erosions and bone edema in 15 bones). Secondly, RAMRIS is a semiquantitative scoring system (e.g., the actual volumes of erosions or edema are not reported), and thus, it may seem too rigid, especially for quantifying early changes or therapy monitoring. A limited set of RAMRIS scores can be also a source of a substantial inter-operator variability [6].

Although a diagnostic standard for assessing RA in MR images of wrist is used for almost a decade, there is currently no commercial tool supporting automated evaluation of synovitis, bone edema and erosions within the wrist joints. In fact, no comprehensive framework for automated evaluation of these lesions has been even published. Although recently there have been some efforts to develop quantitative methods of assessing RA-related changes and comparing them with RAMRIS outcomes [7, 8], these efforts were essentially based on manual outlining of wrist bones or joints borders in 3T MR images of wrist. The authors reported good correlation between RAMRIS and manual outline-based erosion measures, mild correlation between RAMRIS and manual outline-based edema measures, and strong correlation between RAMRIS and manual outline-based measures of synovitis. Although based on manual outlining, the results of Crowley et al. [7] and Chand et al. [8] demonstrated that RAMRIS-based assessment of RA can be possibly replaced with a computer-aided detection (CAD) tool for estimating the volumes of lesions.

Given the aforementioned results, it can be anticipated that automated tools for evaluating RA lesions within wrist joints will emerge in the near future. Indeed, development of CAD systems is a straightforward consequence of introduction of diagnostic standards. For example, after years of research, automated tools for evaluating knee joints in RA have been recently introduced by manufacturers of MR scanners. Due to its anatomy, the case of the wrist joints is obviously much more complicated than the case of the knee joint. Clearly, the most important functionality of the future CAD systems for supporting evaluation of RA within the wrist joints is segmentation of the wrist bones. Segmentation of wrist bones enables subsequent analysis of their interior, i.e., the marrow space, for the search and quantification of bone edema. It also enables detection of joints, i.e., inter-bone regions, for the quantification of synovitis. Finally, comparison of the external surfaces of bones with anatomic models enables detection and quantification of bone erosions.

In spite of its importance, an algorithm for automated segmentation of wrist bones in MR images has not been published to date. There are a few studies focused on segmentation of eight carpal bones in CT [9, 10] or MR images [11], while RA diagnosis requires evaluating 15 wrist bones. More importantly, there is no wrist segmentation framework designed for low-resolution low-field MR images. Recently low-field (0.2T) dedicated extremity MRI scanners became popular primarily because these modalities are considerably less expensive, more comfortable for patients and better accessible than high-field scanners. Low-field scanners offer, however, lower image quality due to lower signal-to-noise ratio and worse resolution and thus dedicated segmentation software must be designed to deal with this kind of data.

In the present paper, we focus on developing a wrist segmentation framework for low-field MR images. Because, for this kind of data, soft tissues like skin and bone marrow are within very similar signal intensity ranges, it is not possible to use methods proposed in other studies [9–11] for CT or high-field MR images. To avoid the risk of false classification of image voxels due to low resolution resulting in weak object edges, we decided to design a framework which simultaneously segments multiple objects. The main contribution of this paper is the development of an algorithm for automated segmentation of 15 wrist bones from MR images. This algorithm forms the base for further research and developments, in particular for the automated detection of synovitis, bone edema and bone erosions, which will be the subject of further studies.

The remaining part of this paper is organized as follows. In “Background on the wrist anatomy” section, the background on wrist anatomy is presented. In “Material and methods” section, we describe details concerning the analyzed MR images and tools used in the present paper. In “Results and discussion” section, the segmentation algorithm is described in detail.

## Background on the wrist anatomy

**Fig. 1:**
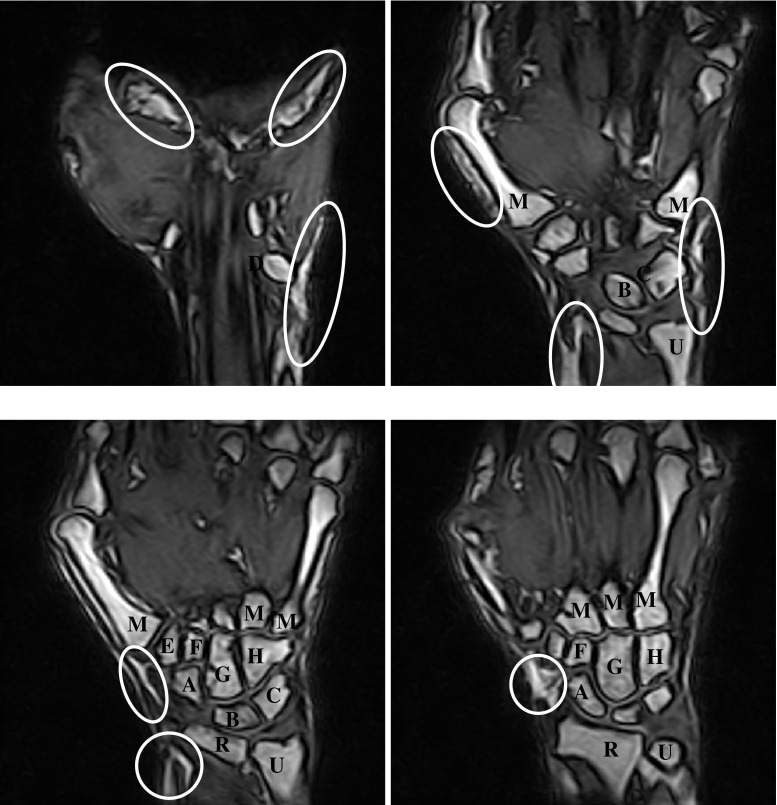
Four coronal slices of MR image of a wrist. *Ellipses* surround soft tissue regions characterized by the same signal intensity range as wrist bone interiors. In the slices, wrist bones are marked: *R* radius, *U* ulna, *M* metacarpals, *A* scaphoid, *B* lunate, *C* triquetrum, *D* pisiform, *E* trapezium, *F* trapezoid, *G* capitate, *H* hamate

A wrist is a complex joint between the distal forearm and the hand. It is composed of 15 bones: distal parts of radius and ulna, eight carpal bones and proximal parts of five metacarpal bones (Fig. 1). All of the bones form various articulations enabling complex mobility of the hand. The carpal bones are organized into a proximal and a distal row. The proximal row includes the scaphoid, lunate, triquetrum and pisiform. The distal row consists of trapezium, trapezoid, capitate and hamate. Numerous ligaments provide stability of the wrist bones and bind carpals to other carpals, to metacarpal bases, to radius or to ulna. The distal row of carpal bones articulates with the bases of the metacarpal bones forming the carpometacarpal joints. Other articulations within the wrist form the radioulnar, radiocarpal and intercarpal joints.

## Material and methods

### MRI data

Thirty-two consecutive patients of the Department of Rheumatology of the author’s institution university hospital participated in the study. Each patient underwent a complete physical examination by the same rheumatologist according to the 2010 ACR/EULAR criteria. Patients had active rheumatoid arthritis (RA) for a duration of at most 5 years. All patients had MRI of at least one wrist as a part of the diagnostic procedure. If RA symptoms were reported in both wrists, MRI of both wrists was performed on two different days. In total, 37 study cases were collected. MRI examinations were performed using a 0.2T extremity E-scan (ESAOTE Ltd, Genova, Italy). The patients were examined in supine position in accordance with the recommendations of the manufacturer. No other special hand-positioning procedures were applied prior to the examination. Four coronal slices of a sample MR image are shown in Fig. 1. Note that basic MR equipment such as 0.2T extremity E-scan does not offer specialized MR sequences enhancing signal from structures of interest (bone in our case), for example fat saturated sequences. For this reason, anatomic structures or tissues (e.g., skin) other than bones are imaged at similar intensity range as bones which poses eventually a serious problem for an automated segmentation of wrist bones.

Among all MRI sequences, acquired for the diagnostic purposes in accordance with the recommendations of OMERACT/EULAR (2), a pre-contrast coronal turbo 3D T1-weighted gradient echo (TR 35 ms, TE 16 ms, pixel spacing 0.75 mm and slice thickness 0.7 mm) was used in the present study. All captured sequences were exported to 16-bit per voxel unsigned raw data files and further analyzed.

### Segmentation quality metrics

Typically, to test the quality of segmentation, the segmentation results are compared with some ground truth data based on some similarity criteria. Frequently, in cases of medical data, the ground truth is generated based on manual segmentation accomplished by a trained individual. In the present paper, to evaluate the proposed algorithm, three similarity criteria between a ground truth and an automated segmentation result were used.

To calculate the area under curve (AUC) of the receiver operating characteristic (ROC), we calculated the number of true-positive (TP), false-positive (FP), true-negative (TN) and false-negative (FN) classifications of voxels. Then, the false-positive rate FPR $=$ FP/(FP+TN) and true-positive rate TPR $=$ TP/(TP $+$ FN) were calculated. Finally, AUC was equal to the area under the ROC, which consisted of three points: {(0, 0), (FPR, TPR), (1, 1)}:

1 $$\begin{aligned} {AUC}=\frac{{FPR}^{*} {TPR}}{2}+(1-{FPR})\frac{1+{TPR}}{2} \end{aligned}$$

An AUC equal to 1 characterizes an ideal classifier while AUC equal to 0.5 is expected for a classifier which assigns cases to classes randomly (with equal probabilities to positive and negative classes). Mean similarity (MS) was defined to measure the region accuracy of segmentation result and ground truth, according to the following formula:

2 $$\begin{aligned} {\textit{MS}}=\frac{\left| {{Im}_{{g},{i}} \cap {Im}_{{s},{i}} } \right| }{\left| {{Im}_{{g},{i}} \cup {Im}_{{s},{i}} } \right| } \end{aligned}$$

where ${Im}_{{g},{i}}$ and ${Im}_{{s},{i}}$ are ground truth and segmented i-th image, respectively, and ${\vert }$.${\vert }$ stands for the number of pixels within an argument image. A MS closer to 1 corresponds to a segmentation result closer to the ground truth.

The mean absolute distance (MAD) was calculated to evaluate the difference between the ground truth boundary and the segmented boundary. MAD was estimated by the means of granulometric analysis [12]. For this purpose, an XORi image was calculated for each image pair ($${Im}_{{g},{i}}, {Im}_{{s},{i}})$$:

3 $$\begin{aligned} {\textit{XORi}}=\left( {{Im}_{{g},{i}} \cap \overline{{Im}_{{s},{i}} } } \right) \cup \left( {{Im}_{{s},{i}} \cap \overline{{Im}_{{g},{i}}}}\right) \end{aligned}$$

where $\overline{{Im}_{.,{i}} }$ denotes negative of ${Im}_{.,{i}}$. Then, a series of morphological openings with a unit diamond structuring element was applied to each XORi. The openings were indexed with an integer *n*, starting from $n=$1. As the result of the procedure, one obtained a series of opened images $\Phi _{{n}}({XOR}_{{i}})$. A granulometric pattern spectrum ${\textit{PS}}_{\Phi }({\textit{XOR}}_{{i}})({n})$ of XORi was equal to [12]:

4 $$\begin{aligned} \forall {n}>0,{PS}_\Phi ({\textit{XOR}}_{i} )({n})=\left| {\Phi _{{n}-1} ({\textit{XOR}}_{i})} \right| -\left| {\Phi _{n} ({\textit{XOR}}_{i})} \right| \end{aligned}$$

Then, we defined MAD as:

5 $$\begin{aligned} {\textit{MAD}}=\frac{\sum \nolimits _{{n}=1} {{n}\cdot {PS}_\Phi ({\textit{XOR}}_i )({n})} }{\sum \nolimits _{n=1} {PS_\Phi (\textit{XOR}_i )(n)}} \end{aligned}$$

With lower values of MAD, the segmentation becomes more accurate, compared to the ground truth.

### Image processing tools

The code of the wrist bones segmentation algorithm utilizes tools provided by ITK library (www.itk.org) and pandore library (https://clouard.users.greyc.fr/Pandore/). In particular, ITK registration framework and pandore implementation of 3D watershed provide the main ingredients of our segmentation package.

### Detection algorithm

**Fig. 2:**
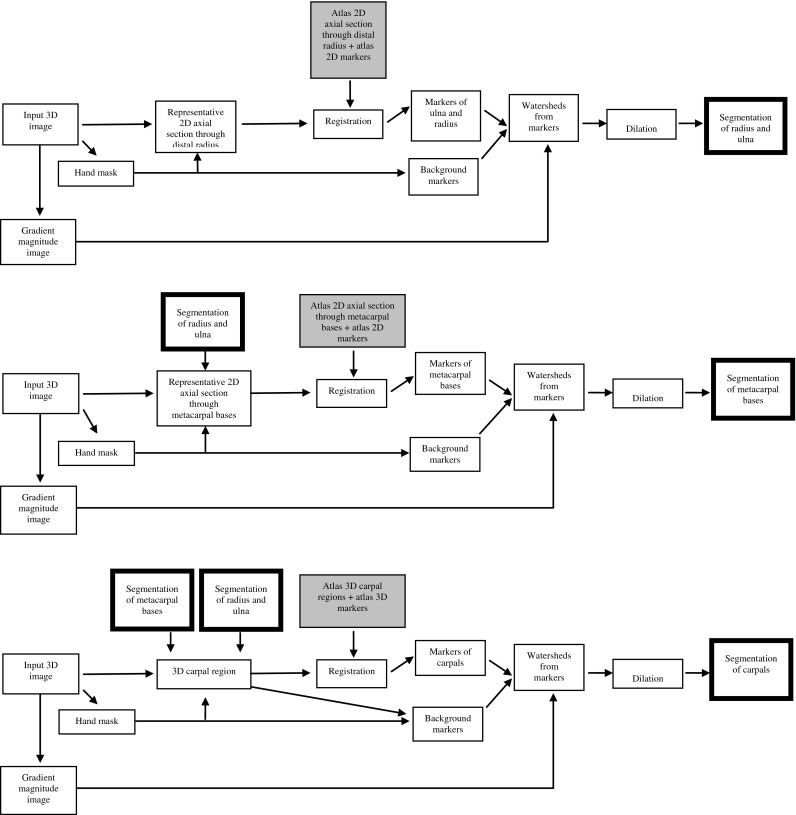
Flowchart of the wrist bones segmentation algorithm: *boxes* with *gray* background and *thin borders* denote atlas input, *boxes* with *white* background and *thick borders* denote partial segmentation results, *boxes* with *gray* background and *thick borders* denote final segmentation results, a *box* with *black* background denote a step in which user interaction is required

The flowchart diagram of the wrist bones segmentation algorithm is presented in Fig. 2. The core of the algorithm is the atlas-based segmentation, which requires registration of an actual image with an atlas image. This is a natural choice since the anatomy of analyzed objects is fixed to a large extent. Because registration in three dimensions is computationally expensive, we chose to divide the segmentation problem into subproblems. Thus, the algorithm consists of four modules: segmentation of the hand mask, segmentation of the distal parts of the ulna and radius, segmentation of the metacarpal bases and segmentation of carpals. We use registration in all steps apart from mask segmentation: In the first two cases, we use a relatively cheap 2D algorithm and a 3D algorithm in the third step. The segmentation procedure uses the “watershed from markers” algorithm [13]. At the input of this algorithm, one must provide a magnitude of gradient image and a marker image. The gradient image can be interpreted as a topographic relief, where the gradient value at a voxel is equivalent to its altitude in the relief. The watershed of a relief corresponds to the limits of the adjacent catchment basins, and each catchment basin contains exactly one marker. The gradient components were calculated using Prewitt’s filter for an original MR image blurred with a Gaussian filter with fixed half-size equal to 1 voxel. The atlas-based segmentation provides a method for automated selection of markers, enabling robust watershed-based segmentation of the wrist bones. The markers constructed by our algorithm can be an input for other procedures (e.g., region growing or deformable models) but given the results of the evaluation of watershed-based segmentation, we expect in such cases only marginal if any improvement of the segmentation results.

#### Segmentation of the mask

The purpose of the mask segmentation was to extract from the field of view the regions corresponding to an examined hand. This step consisted of two substeps:

1. Thresholding an original MR image with automatically selected threshold,
2. 2D morphological processing of all axial slices of an original MR image.

**Fig. 3:**
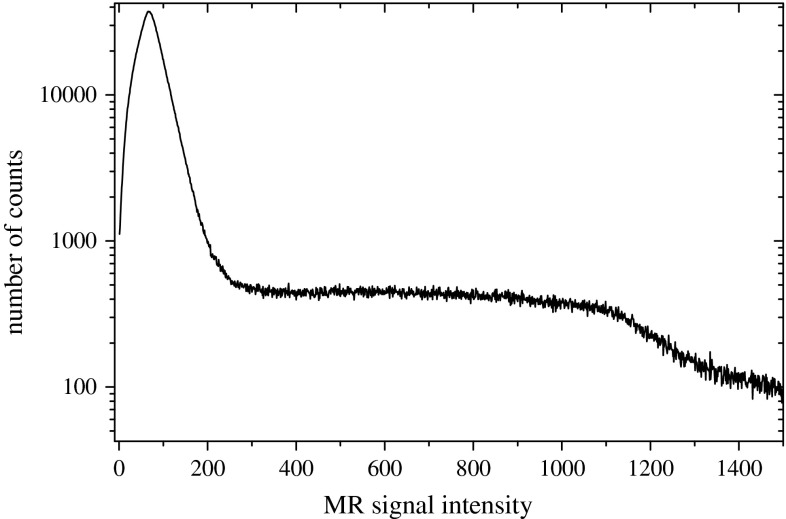
Histogram of *gray* levels of MR image of wrist

The segmentation of the mask started from thresholding an original MR image. The threshold TH was selected based on the analysis of histogram of signal intensities (Fig. 3). In all cases, the histogram consisted of a large mode at low intensities corresponding to image regions surrounding an examined hand and a broad mode for higher intensities corresponding to anatomic structures. The histogram was smoothed with a running average (box equal to 11 gray-levels), and the threshold TH was set at the minimum of the histogram right to the leftmost mode. Because this minimum was always located in a range from ${I}_{{\textit{LOW}}} = 200$ to ${I}_{{\textit{HIGH}}} = 500$, the search for TH was restricted to this range only.

**Fig. 4:**
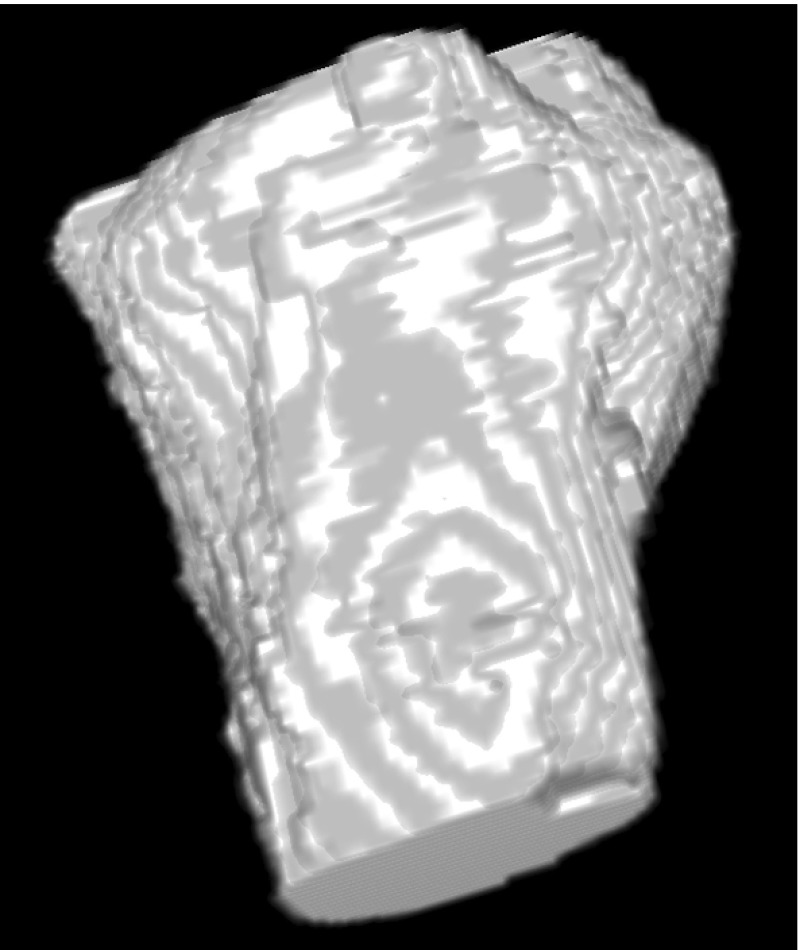
3D visualization of a hand mask

Next, for every 2D axial slice of a segmented 3D image, we applied morphological closing with a square structuring element (half-size BOX equal to 5 pixels) followed by 2D hole filling. Finally, the processed 2D axial slice was eroded with a square structuring element (half-size equal to BOX/2 pixels). A sample visualization of a hand mask is shown in Fig. 4. The surface of this mask was within external regions of the hand and thus could be used as a marker of soft tissues characterized by signal intensity in the same range as the wrist bone interiors.

Because we were not interested in accurate reconstruction of the external hand surface but in creating a soft tissue marker, detailed tuning of TH was not very important. The values of ${I}_{{\textit{LOW}}}$ and ${I}_{{\textit{HIGH}}}$ were fixed for all samples because non-tissue regions were always imaged within similar intensity ranges, and only marginally influenced by the actual composition of hand tissues. Two-dimensional morphological processing was chosen instead of a 3D one, because it resulted in faster processing and lower roughness of the external mask surface with fewer cavities. The morphological closing was necessary to include tissues characterized by low MR signal in the mask, i.e., cortical bone or tendons. The full size of the structuring element (11 pixels or about 8mm) was selected to be larger than typical thickness of these structures. Because we did not want the external surface of the mask to cross bones, the half-size of the final erosion (2 pixels or about 1.5mm) was selected to be smaller than typical thickness of skin tissue.

#### Segmentation of the distal parts of ulna and radius

The segmentation of the ulna and radius consisted of the following steps:

1. Automatic selection of a representative cross section through the ulna and radius within an analyzed image,
2. Registration of the representative cross section through the ulna and radius to an atlas cross section with simultaneous transformation of an atlas marker to the space of the representative cross section,
3. 3D marker preparation based on the results of registration,
4. Watershed from marker segmentation of the ulna and radius followed by the final processing.

**Fig. 5:**
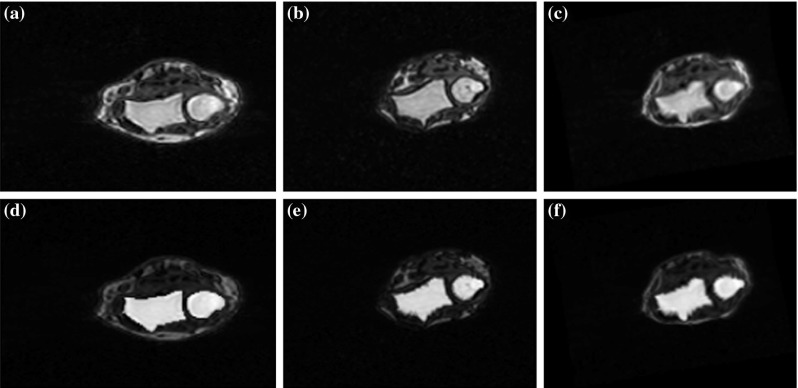
**a** Atlas, **b** sample, and **c** registered atlas axial cross sections through ulna and radius. **d**–**f** The same as above with markers superimposed

**Fig. 6:**
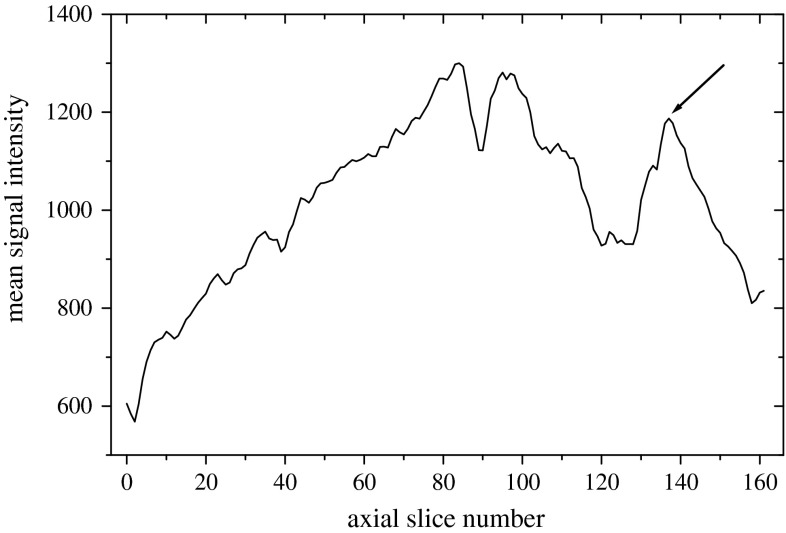
Mean signal intensity in axial cross sections plotted versus axial slice number. Low values of the axial slice number correspond to metacarpal regions and high values—to distal parts of radius and ulna. An axial slice selected for as a fixed image of the registration is marked with an *arrow*

Proper selection of a representative radius and ulna axial cross section for the registration procedure (Fig. 5b) was crucial for successful segmentation of these bones. To select this cross section, we built a classifier based on the mean MR signal intensity profile within axial cross sections of the MR image (Fig. 6). In the figure, low values of the axial slice number correspond to the metacarpal regions, while high values correspond to distal parts of radius and ulna. The calculations of the mean intensity were limited only to the hand mask voxels. Consequently, one can expect a high mean intensity value for a slice with the highest ratio of the number of ulna and radius pixels to the number of the mask pixels. Moving to the lower values of the axial slice number, one finally gets into a minimum of the profile corresponding to the radioulnar joint region.

The radius and ulna axial cross section for the registration procedure were selected at the first maximum from the right of the plot such that there was a sufficiently deep minimum (i.e., we required that the relative difference between minimal and maximal values, computed as: (maximum intensity $-$ minimum intensity)/(0.5 * (maximum intensity $+$ minimum intensity)), is larger than 10 %) left to the maximum at sufficiently close distance (less than 15 % of the axial extent of the field of view). This classifier was build based on a training set consisting of a half of our image database and then tested on the other half of images. This classifier required only two parameters to accomplish segmentation of the ulna and radius in all cases.

A core of the algorithm of the segmentation of the ulna and radius is a registration of an atlas axial cross section MR image of these bones and a representative axial cross section of an examined MR image. For an atlas cross section, we prepared a corresponding binary atlas marker image with manually outlined markers corresponding to the radius and ulna bones (Fig. 5). When the tested case had a different orientation than an atlas image, the atlas and marker images were flipped accordingly.

The registration was accomplished using the ITK registration framework. The ITK registration framework requires specifying a few components: a moving image (the atlas axial cross section), a fixed image (an axial cross section selected from an analyzed image), a transform component representing the spatial mapping of points from the fixed image space to points in the moving image space, an interpolator used to calculate moving image intensities at non-grid positions, a metric providing a measure of how well the fixed image is matched by the transformed moving image and an optimizer which uses a metric to search for an optimal matching of moving and fixed images in the space defined by the parameters of the transform. In the calculations, we have used a linear interpolator, regular step gradient descent optimizer and mean square image-to-image metric. Because a fixed and a moving image can be shifted and rotated relative to each other, to make the registration robust, we applied a sequence of transformations starting from a rigid transform, then an affine transform and finally a coarse-grid and a fine-grid deformation transform. After completing the registration, the final transform parameters were used to map an atlas marker image to the space of the fixed image, resulting in ulna and radius markers for a given case. The marker image after transforming it to the fixed image space was no longer binary, and thus, it was thresholded with threshold equal to 1 (i.e., all nonzero gray values were replaced by a single value corresponding to the foreground). In rare cases of large deformations necessary to transform a moving image into a fixed image, a single marker in an atlas marker image was transformed to a few disjoint parts. Thus, after thresholding, only two largest components of the transformed marker image were eventually left for further analysis, resulting in 2D candidate markers.

The atlas axial cross section through the ulna and radius, an axial cross section from a sample analyzed image and the atlas cross section registered to a sample cross section are shown in Fig. 5. Binary markers superimposed on the cross section images are also shown in the figure.

To stabilize the 3D watershed, 2D candidate markers constructed based on registration were extended to 3D by means of a region growing procedure. Firstly, the voxels at the center of mass of the 2D candidate markers were determined and set as the seeds for a region growing procedure. Next, the seeds merged image voxels in the order of decreasing MR signal intensity. The growth of the clusters was restricted to the proximal direction and was controlled by the breadth-first search strategy. It was stopped when a first voxel $V$ at a distance $D =$ 10 slices (about 7 mm) from the seed was acquired. Then a path of voxels connecting $V$ to the seed $S$ was reconstructed, representing how $V$ was reached from the seed $S$ during the growing process (i.e., this path is a sequence of voxels {$V, {V}_{1}, {V}_{2}$, ..., ${V}_{{n}}, S$}, such that ${V}_{1}$ is a neighbor of $V$ and $V$ was merged when the neighborhood of ${V}_{1}$ was analyzed during the growth process, ${V}_{2}$ is a neighbor of ${V}_{1}$ and ${V}_{1}$ was merged when the neighborhood of ${V}_{2}$ was analyzed during the growth process, ..., ${V}_{{n}}$ is a neighbor of S and ${V}_{{n}}$ was merged when the neighborhood of $S$ was analyzed during the growth process). This path together with the seed formed the final 3D marker of either the ulna or radius. The distance $D$ was selected to be close to the typical extent of the radioulnar joint. Radius and ulna markers, together with a background marker constructed from the surface of the hand mask (as described in detail in the previous subsection), formed a set of markers necessary for the 3D watershed-based segmentation of ulna and radius.

Because of the physical background of MR imaging, cortical bone appeared darker than neighboring internal marrow and external joint regions. Consequently, in the gradient magnitude images, there were ridges corresponding to both the internal and external surfaces of the cortical bone. Because of this, watershed from markers segmented marrow regions while cortical parts of bone were not included in the segmentation. Thus, in the final step of the ulna and radius segmentation, we applied morphological dilation with a diamond structuring element (half-size equal to 1 voxel, about 0.7 mm). The size of the structuring element was selected to be close to the typical thickness of cortical bone within the wrist bones.

#### Segmentation of the metacarpal bases

The segmentation of the metacarpal bases was analogous to the segmentation of the radius and ulna, that is, an atlas axial cross section MR image of these metacarpal bases was registered with a representative axial cross section through metacarpal bases of an examined MR image. The only difference was in selecting the representative cross section through the metacarpal bases to be registered with the atlas cross section. The task of selecting the representative cross section consisted of the following sub-tasks:

1. Thresholding the original MR image;
2. Filtering voxel clusters based on a properly selected feature set;
3. Selection of the representative cross section based on the restricted number of clusters.

**Fig. 7:**
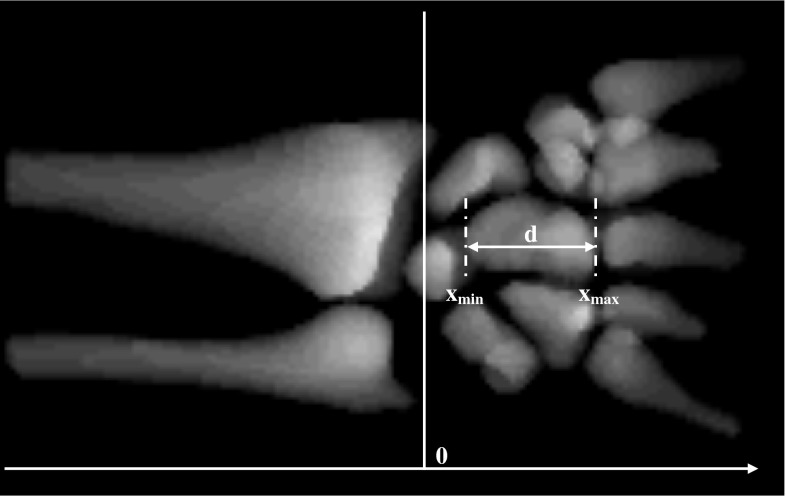
Cluster features used to filter connected components prior to selection of a representative cross section through metacarpal bases

Firstly, the original MR image was thresholded with threshold ${ TH}_{{\textit{MCP}}} = { TH}_{{\textit{ME}}}+ \textit{BUFFER}$, where ${ TH}_{{ME}}$ was determined by the maximum entropy algorithm [14] and BUFFER was a constant. The value of BUFFER was selected large enough to separate clusters corresponding to different bones and was equal to 200 in all cases. Next, for each cluster, a number of features was calculated including the extent d in the axial direction, the minimum ${x}_{{min}}$ and maximum ${x}_{{max}}$ coordinate in the axial direction (assuming origin of the reference frame at the apex of the styloid process, axial direction along the axis of the radius or ulna with positive values in the distal direction-see Fig. 7 for further explanations). Because clusters corresponding to metacarpals are typically large and well separated from the distal radius, only $N$ clusters with the largest extent were left such that their ${x}_{{min}}$ was higher than some threshold value ${x}_{{\textit{TH}}}$. It was found that $N$ equal to 10 and ${x}_{{\textit{TH}}}$ equal to 0.35 *L (where L is a linear extent of the field of view in the axial direction) are sufficient to filter out carpals and leave all metacarpals in all cases. Finally, the axial slice in the filtered binary image with the highest average intensity was selected as the representative cross section through the metacarpal region.

**Fig. 8:**
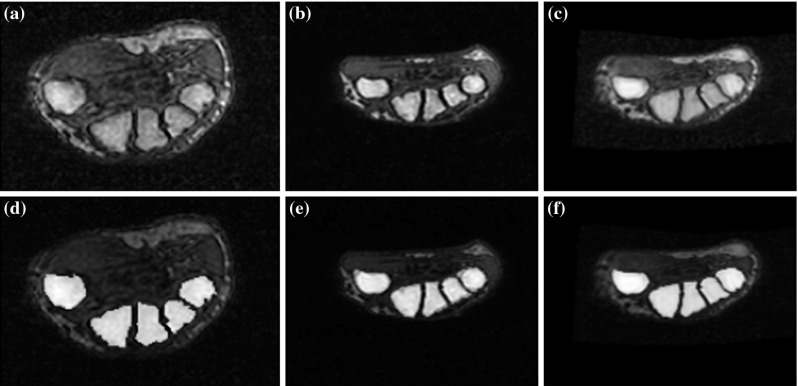
**a** Atlas, **b** sample, and **c** registered atlas axial cross sections through metacarpal bases. **d**–**f** The same as above with markers superimposed

The atlas axial cross section through metacarpal bases, an axial cross section from a sample analyzed image and the atlas cross section registered to a sample cross section are shown in Fig. 8. Binary markers superimposed on the cross section images are also shown in the figure. As in the case of the radius and ulna, the transformed marker image was thresholded with threshold equal to 1 and five largest components of the transformed marker image were left for further analysis.

Note that because of a great inter-patient variability of sizes of metacarpal bases, it is possible that the borders of the markers overlap with bone boundaries, after mapping markers from the space of the atlas image to the space of the fixed image. To avoid such problems, after registration, each of the five markers of metacarpal bases was replaced by its central pixel (defined as the pixel at the largest distance from the background). Next, each one-pixel marker was extended to 3D in the distal direction analogously as in the case of the radius and ulna markers. These markers, together with a background marker constructed from the surface of the hand mask, formed a set of markers necessary for the 3D watershed-based segmentation of metacarpal bases.

#### Segmentation of the carpals

The segmentation of carpals consisted of the following steps:

1. Automatic selection of the 3D carpal region within an analyzed image,
2. 3D registration of the carpal region to an atlas carpal region with simultaneous transformation of an atlas marker to the space of the analyzed image,
3. 3D marker preparation based on the results of registration,
4. Watershed from marker segmentation of carpals followed by the final processing.

**Fig. 9:**
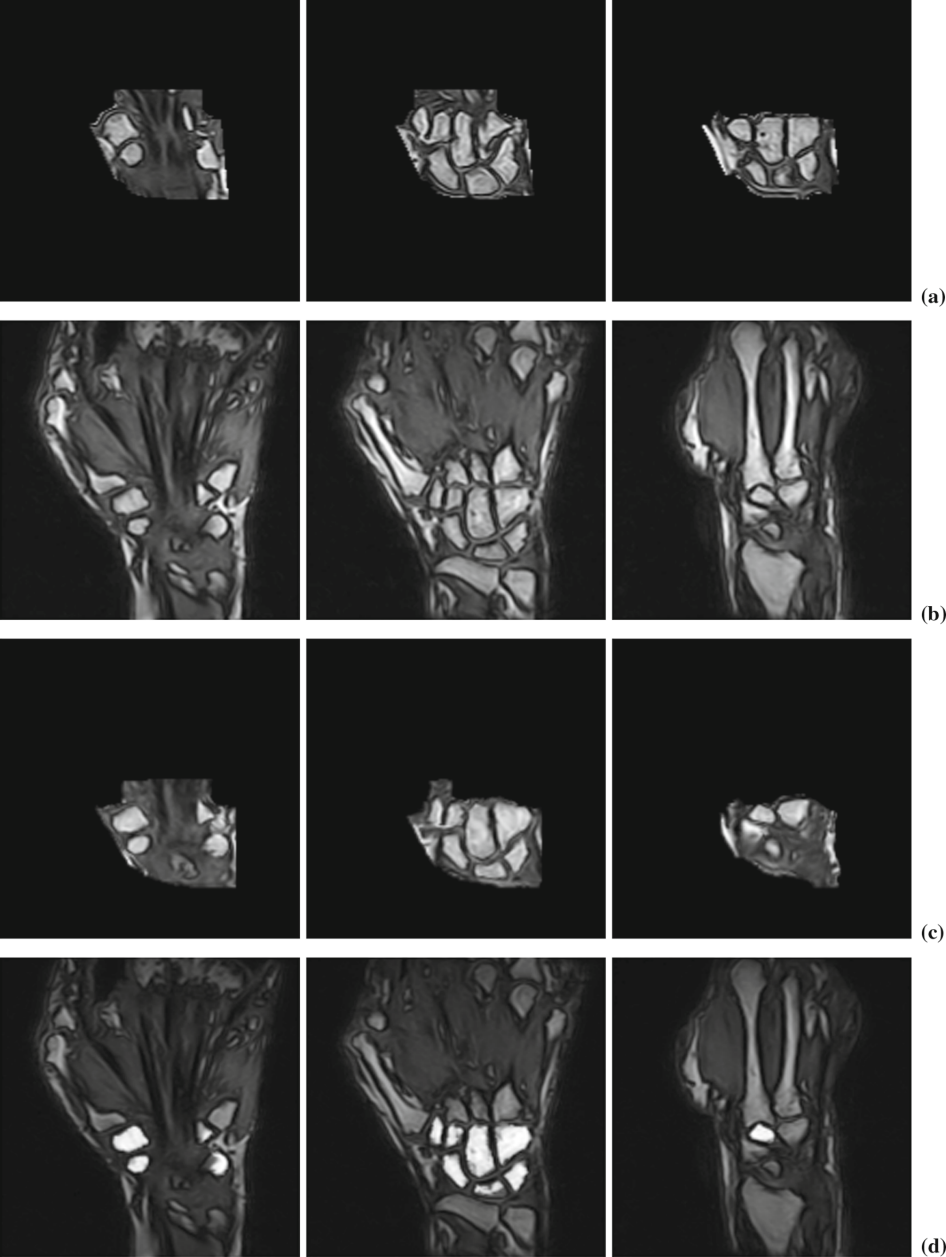
Coronal slices from MR images of wrist: **a** an atlas image, **b** a sample image, **c** and atlas image registered atlas to a sample image, **d** atlas markers transformed to the space of the sample image

Prior to the segmentation of carpals, a 3D atlas image of the carpal region was prepared together with a corresponding marker image. Based on the results of segmentation of the ulna, radius and metacarpal bases, a 3D region containing the carpals was determined within an analyzed image. In particular, to find the carpal region, we determined the distal envelope of the ulna and radius and the proximal envelope of the metacarpal bases. Then, two planes were found which bounded the carpal region from the right and from the left, one tangential to the ulna and the base of fifth metacarpal, and the other one tangential to the radius and to the first metacarpal base (Fig. 9a).

An atlas 3D carpal region (moving 3D image) was registered with an analyzed carpal region (fixed 3D image) and, based on the parameters of the final transform, 3D atlas markers were transformed to the space of the fixed 3D image resulting in a candidate markers image. A few atlas slices of the carpal region, slices of the carpal region from a sample analyzed image, the atlas carpal region registered to a sample carpal region and slices of the sample carpal region with markers superimposed are shown in Fig. 9. Note that although the atlas and the sample images are quite different, the atlas markers transformed to the space of the sample image fit very well to the interiors of the carpals.

**Fig. 10:**
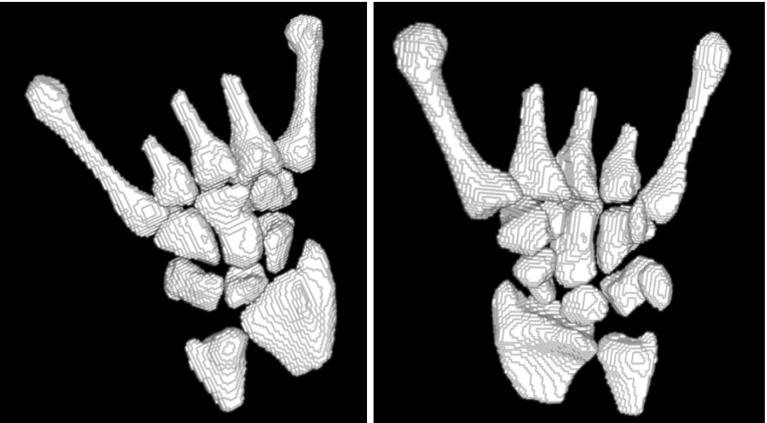
Sample views of a 3D segmentation of wrist bones

Because candidate markers can intersect the borders of the carpals in the analyzed image, some processing was necessary to convert them into the final markers. Firstly, because the gray values of the marker image cover an 8-bit range after transforming it to the space of the fixed image, the candidate marker image was thresholded with a threshold equal to 240. Secondly, to assure that marker voxels are within bright regions of an analyzed image, a threshold ${ TH}_{{ME}}$ was calculated for an original MR image, based on the maximum entropy algorithm. Then, all marker voxels, for which MR signal intensity in corresponding voxels in the original MR image was lower than ${ TH}_{{ME}}+\textit{BUFFER}$, were converted to background voxels. In the calculations, we used fixed value BUFFER $=$ 200. Next, morphological closing (square structuring element, half-size equal to 1 voxel) and hole filling were applied to every axial slice of the binary image of markers. Finally, only eight largest connected components were left in the marker image resulting in final markers of the carpals. The background marker consisted of all analyzed image voxels which were not within the carpal region. These markers formed a set of markers necessary for the 3D watershed-based segmentation of carpal bases. A sample segmentation of all wrist bones is shown in Fig. 10. Note that some metacarpals are only partially segmented. The algorithm was, however, designed to segment metacarpal bases (which are evaluated in RA diagnosis) but not to segment metacarpal diaphyses.

## Results and discussion

The approach for segmentation of bones in magnetic resonance images of the wrist proposed in this paper can be rated on the basis of test quality, computational cost and its limitations.

We used five different sets of atlas images for each step to test the sensitivity of the algorithm to atlas and marker image selection. The atlas images were selected from five randomly selected cases. The choice of atlas slices from the radioulnar image region was guided by the requirement of the largest axial cross-sectional area of radius and ulna (Fig. 5). Similarly, the atlas axial cross sections through metacarpal bases were based on the largest axial cross section area of the bases (Fig. 7). Finally, the atlas images of wrist bones were three-dimensional (Fig. 9), and thus, no special selection procedure was required (besides masking no-wrist region). The markers were outlined manually based on expert-chosen slices from original cases and covered either the cross section or volumes of the bones. For all sets in each step, we obtained properly segmented bones with minor discrepancies. For each step and case, we also prepared a referential image that consisted of pixels chosen by majority voting among five segmentations resulting from different markers. We subtracted it from segmentations resulting from different markers and calculated the average and standard deviation for the subtractions. These values are shown in Table 1. The results show that the algorithm is independent from atlas image.

**Table 1:** The average (standard deviation) of the subtraction of majority voting result image and segmentations based on different masks for all steps

	Marker 1	Marker 2	Marker 3	Marker 4	Marker 5	All Markers
Radius and ulna segmentation	0.001 (0.004)	0.0001 (0.0003)	0.003 (0.11)	0.0001 (0.0002)	0.01 (0.00)	0.001 (0.005)
Metacarpal bases segmentation	0.002 (0.001)	0 (0)	0 (0)	0 (0)	0.000005 (0.00001)	0.00005 (0.0001)
Carpals segmentation	0.02 (0.07)	0.03 (0.06)	0.03 (0.09)	0.0008 (0.0009)	0.008 (0.04)	0.02 (0.06)

To test the quality of the proposed segmentation algorithm, we conducted manual segmentation of all thirty-seven cases. The results of the manual outlining were treated as ground truth. Table 2 summarizes the average and standard deviation values of the area under the ROC (AUC), mean similarity (MS) and mean absolute distance (MAD) between manual and automated segmentation in three groups: distal parts of radius and ulna, metacarpal bases and carpal bones. The average and standard deviation values AUC, MS and MAD between the manual and automated segmentation majority voting images are shown in Table 3. The results prove the correctness of the proposed method as compared to manual segmentation. The object borders, as detected by watersheds from markers, are localized at object boundaries. The values of MAD (which are very close to one) indicate that the distance between the contours of automated and manual segmentations is approximately equal to 1 pixel. For comparison, in the study of Koch et al. [11] (which was based on MR images with two times higher resolution than in the present study), the segmentation accuracy was equal to 0.48 mm, compared to 0.365 mm voxel size. For the same study, the values of AUC do not exceeded 0.91.

**Table 2:** The average (standard deviation) of area under ROC (AUC), mean similarity MS and mean absolute distance MAD between automated segmentation and manual segmentations performed on 3D images in three groups: distal parts of radius and ulna, metacarpal bones and carpal bones

	AUC	MS	MAD [voxel]
Radius and ulna segmentation	0.99 (0.02)	0.99 (0.08)	1.10 (0.21)
Metacarpal bases segmentation	0.98 (0.05)	0.93 (0.07)	1.15 (0.25)
Carpals segmentation	0.95 (0.08)	0.89 (0.12)	1.45 (0.36)

**Table 3:** The average (standard deviation) of area under ROC (AUC), mean similarity MS and mean absolute distance MAD between automated segmentation and manual segmentations performed on 3D images

AUC	MS	MAD [voxel]
0.97 (0.04)	0.93 (0.09)	1.23 (0.28)

Concerning the computation time (Pentium Dual Core, 2.6 GHz, 3.25 GB RAM, single core was used in the computations), the most demanding part of the segmentation was the 3D registration of an atlas and an actual carpal regions, which frequently took even up to half an hour to complete. The registered regions were typically about $100\times 100\times 60$ voxels in size. The registration, based on ITK implementation, was driven by the mean square image-to-image metric, which required computation of signal intensity differences between all corresponding voxels in a moving and a fixed image. This was the most computationally expensive part of the registration. Other ITK image-to-image metrics produced much worse registration results although in a shorter time. The other parts of the segmentation procedure were not as expensive as 3D registration. The second-most expensive procedure was 3D watershed from markers which took not more than about 1–2 min for full-size images (typically about $160\times 160\times 120$ voxels). Total computation time, excluding 3D registration, was never longer than about 5 min.

In this study, over 500 bones were segmented (37 cases times 15 bones per a case) and there were no significant deviations from gold-standard and automated segmentation (as demonstrated in Table 2). The main limitation of our algorithm is that it is sensitive to medium and large bone erosions (in which case the manual segmentation is also problematic, given low quality, low-field MR data). In the case of small erosions, the algorithm behaves correctly; all fifteen bones are detected. In the case of large erosions, we can distinguish two types of flaws: missing segments or a cloud of unrelated points or distorted segments. Because small erosions are typical for early stages of RA, our algorithm may be used for early diagnostics without any changes. To overcome this limitation, we plan to use registration with several atlas images altogether with deformable models on the best fit to model the erosion and estimate its stage. Of course, in especially difficult cases, the algorithm can be easily integrated with manual or semi-manual procedures to correct for erroneous classification of image voxels. In fact, as in all medical procedures, the final decision must be left to a qualified medical personnel. However, it should be noted that MRI is especially valuable when diagnosing early stages of RA, when erosions are not present. Then, the proposed algorithm can be the first stage for the detection of early lesions like bone edema or synovitis.

The results of the present study demonstrate that automated segmentation of wrist bones is feasible even in the case of as low image quality as in the case of 0.2T images. Further research can proceed along a few ways. Firstly, we have proposed an algorithm for automated markers selection. The markers can be used by other segmentation procedures starting from markers, not necessarily watersheds, e.g., region growing, geodesic contours. Finally, given the segmentation results, an automated diagnostic system for quantification of synovitis and bone edema should be developed.
